# Effect of Soy Wax/Rice Bran Oil Oleogel Replacement on the Properties of Whole Wheat Cookie Dough and Cookies

**DOI:** 10.3390/foods12193650

**Published:** 2023-10-02

**Authors:** Aditi Pradhan, Arfat Anis, Mohammad Asif Alam, Saeed M. Al-Zahrani, Maciej Jarzebski, Kunal Pal

**Affiliations:** 1 Center for Biotechnology, School of Pharmaceutical Sciences, Sikha ‘O’ Anusandhan Deemed to be University, Orissa 751030, India; aditipradhan727@gmail.com; 2 Department of Biotechnology and Medical Engineering, National Institute of Technology Rourkela, Odisha 769008, India; 3 SABIC Polymer Research Center (SPRC), Department of Chemical Engineering, College of Engineering, King Saud University, P.O. Box 800, Riyadh 11421, Saudi Arabia; szahrani@ksu.edu.sa; 4 Center of Excellence for Research in Engineering Materials (CEREM), College of Engineering, King Saud University, P.O. Box 800, Riyadh 11421, Saudi Arabia; moalam@ksu.edu.sa; 5 Department of Physics and Biophysics, Faculty of Food Science and Nutrition, Poznań University of Life, Sciences, Wojska Polskiego 38/42, 60-637 Poznań, Poland; maciej.jarzebski@up.poznan.pl

**Keywords:** oleogel, soy wax, dough, cookie, texture analysis, whole wheat flour

## Abstract

This study investigated the replacement of butter with soy wax (SW)/rice bran oil (RBO) oleogel in varied proportions in cookie dough and the resulting cookies. The study mainly evaluates the physical, textural, and chemical properties of the butter cookie dough and cookies by replacing butter with SW/RBO oleogel. The dough was assessed using moisture analysis, microscopy, FTIR Spectroscopy (Fourier Transform Infrared) and impedance spectroscopies, and texture analysis. Micrographs of the dough showed that D-50 (50% butter + 50% oleogel) had an optimal distribution of water and protein. D-0 (control sample containing 100% butter) showed the lowest impedance values. Moisture content ranged between 23% and 25%. FTIR spectroscopy suggested that D-50 exhibited a consistent distribution of water and protein, which CLSM and brightfield microscopy supported. Texture analysis revealed that the dough samples exhibited predominantly fluidic behavior. As the amount of oleogel was raised, the dough became firmer. The prepared cookies showed a brown periphery and light-colored center. Further, a corresponding increase in surface cracks was observed as the oleogel content was increased. Cookies moisture analysis revealed a range between 11 and 15%. Minute changes were observed in the texture and dimensions of the cookies. In summary, it can be concluded that replacing butter with oleogel by up to 50% seems to be feasible without significantly compromising the physicochemical properties of cookie dough and cookies.

## 1. Introduction

Healthy food consumption and delicious flavors are often seen as opposing goals. However, it is possible to achieve health and taste simultaneously. The rise of Western diets and industrial urbanization have significantly contributed to the development of baked goods. Bakery products are in high demand due to their convenience and affordability. Bakery products comprise flour, sugar, butter, eggs, flavoring agents, and salt, making them delicious yet unhealthy. Compared to all-purpose flour, which has a balance between the protein content for structure and tenderness, refined wheat flour (RWF) is calorie-dense (~455 cal/100 g) and has a lower protein content (10.3%) and dietary fiber content (2.7%) [1]. A lower quantity of dietary fiber makes RWF difficult to digest. Furthermore, butter contains significant levels of saturated fatty acids (SFAs), trans fatty acids (TFs), and polyunsaturated fatty acids (PUFAs), all of which can raise cholesterol levels and increase the risk of heart disease [2,3]. As per WHO guidelines, individuals are encouraged to limit their fat intake to less than 30% of total energy consumption, saturated fats to less than 10%, and trans fats to less than 1%. Globally, a discernible change has occurred toward a healthier and more sustainable diet over the past decade. Several factors, such as environmental concerns, animal welfare, and individual health, are behind this shift [4].

Among bakery products, the biscuit segment is the most dominant type, which includes cookies, salt crackers, and cream biscuits [5]. In India, the bakery market is dominated by cookies, which account for about 72% of the market. Cookies are typically made of RWF, sugar, shortening, and salt. They are tiny, sweet baked goods. The type of flour is essential for tailoring the texture, structure, and flavor of cookies. The perfect cookie requires flour, which is a necessary ingredient. It can be made healthier by substituting whole wheat flour (WWF) for refined white flour. This is explained by the fact that WWF is a nutritious food option because it contains many essential nutrients, including fiber, vitamins, and minerals. Furthermore, due to its high dietary fiber content, it is easily digested by humans [6]. Due to the massive consumption of WWF in India, adding it to baked goods can be readily accepted and assimilated into people’s diets. Another essential ingredient is shortening, a form of solid fat commonly used in cookies [5]. Because shortening has a high melting point, it remains solid at room temperature and can resist high temperatures without burning or deteriorating. It improves the flavor and texture of biscuits. Furthermore, it provides benefits by assisting in aeration and stability, which will enhance the overall structure and shape of the product. As a result, it is ideal for making flaky, soft cookies. The most common shortenings include margarine, butter, vegetable shortening, palm oil, and lard. Among these, butter is the most commonly utilized shortening in various items. It is high in fatty acids, which can elevate serum cholesterol levels and increase the risk of heart disease (total fat: 80%, Solid Fatty Acid (SFA): 2%, and trans fat: 0.5%). It has also been linked to an increased chance of type 2 diabetes and some types of cancer [7]. With concerns growing about the health impacts of butter, people are increasingly seeking healthier alternatives for shortening used in cookies and other bakery products. They must resemble butter in terms of functional qualities and texture. Some common substitutes for butter include vegetable oil, olive oil, margarine, lard, ghee, and others [8]. However, their consumption is linked to several health concerns. For instance, consuming large amounts of partially hydrogenated palm oil increases the risk of cancer and heart attack [9]. Oleogels can replace shortening in these circumstances while still being healthy because of their resemblance to butter in texture and low health risks. Oleogels are complex micro-structured systems in which oil is entrapped within a 3D network of oleogelators. Commonly employed oils for developing oleogels include vegetable oils such as peanut oil [10], rice bran oil (RBO) [11], and sunflower oil [12,13]. The oleogelator encompasses a range of substances, such as fatty acids, waxes, fatty alcohols, glycerides, and polysaccharides [6].

**Scheme 1:**
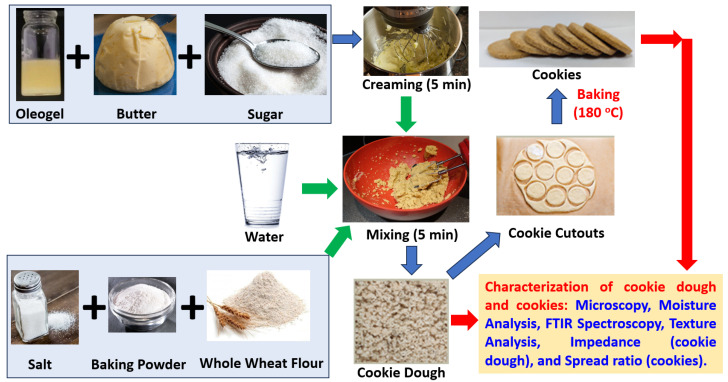
The proposed scheme of the study.

In this study, we substituted the shortening (in this case, butter) in varied concentrations using the optimized oleogel (RBO/SW) formulated by our group, reported elsewhere [14], for the preparation of WWF cookies. RBO demonstrates superior oxidative stability compared to other cooking oils while being a rich source of γ-oryzanol. Similarly, SW is associated with numerous benefits. However, its current use has been limited in the food industry due to its low melting point. It is expected that this approach will aid in the development of healthier cookies and mitigate health concerns. To thoroughly evaluate the impact of incorporating oleogel into cookie dough, we have examined the physicochemical parameters via several methods, including microscopy (bright field and CLSM), moisture content analyzer, FTIR spectroscopy, texture analysis, and impedance analysis. Cookie samples were evaluated via microscopy (surface topology analysis using stereo zoom microscope), moisture analysis, FTIR spectroscopy, and texture analyzer (3-point bending test and puncture test). The summary of the proposed work study has been provided in Scheme 1.

## 2. Materials and Methods

### 2.1. Materials

SW (Tattvalogy, New Delhi, India) and RBO (Fortune Rice Bran Health, Mumbai, India), were used to prepare oleogel. The following ingredients were purchased from neighborhood supermarkets: WWF (Aashirvaad, Mumbai, India); sugar and salt (Tata Chemicals Ltd., Mumbai, India); butter (Amul Pvt. Ltd., Anand, India); and baking powder (Weikfield Food Pvt., Pune, India). According to the information provided on the packaging, the nutritional composition of WWF per 100 g is carbohydrates—76.8 g, energy—340 kcal, protein—10.5 g, total fat—1.4 g, and sodium—2.3 g. RBO comprised fat (saturated fatty acid—24 g, mono-unsaturated fatty acid—40 g, polyunsaturated fatty acid—34 g, trans fatty acid—2 g), vitamin E—50 mg, and gamma oryzanol—1000 mg. The composition of butter had a total fat content of—80 g where (SF—48 g, TF—0.5 g), cholesterol—220 mg, and protein—1 g, while the composition of baking soda was provided as energy—183 Kcal, carbohydrates—45.6 g, protein—0.1 g, sodium—42 g.

### 2.2. Methods

#### 2.2.1. Preparation of Oleogel

Based on the procedure outlined by Dhal et al. (2023), the oleogel was prepared. In brief, 20 g of oleogel was made by combining 3 g of soy wax (SW) and 17 g of rice-bran oil (RBO) in a beaker and placing it in a water bath (65 °C) for 15 min. The mixture was then stirred with a magnetic stirrer for 20 min at 300 rpm to form a homogeneous solution. Then, the beaker was incubated in an incubator for an hour at (25 °C) to induce oleo-gelation. It was then refrigerated at (4 °C) for future usage.

#### 2.2.2. Cookie Dough and Cookies Preparation

##### Cookie Dough Preparation

The ingredients listed in Table 1 were used to make the cookie dough. At first, an electric blender (Model: OHM-207,150 -Watt, Orpat, New Delhi, India) was used for creaming the butter/oleogel (in varied proportions) and sugar mixture at the lowest speed of the blender (speed—1; time—5 min). Then, the flour was gradually added to the mixture along with other dry ingredients (salt and baking soda) and was mixed for another 5 min while adding the water. This resulted in the formation of a dough with a crumbly texture. The prepared dough (named D-0, D-25, D-50, D-75, and D-100) was then sealed with cling film and was refrigerated for 1 h at 4 °C. Thereafter, the dough was converted into a thin sheet (thickness of ~3 mm), which was then cut into circular pieces (dough cutouts) using a cookie cutter (diameter: 50 mm). The average weight of the cut dough cutouts is presented in Table 1.

##### Preparation of Cookies

For cookie preparation, the oven (Model: MC32J, Samsung, South Korea) was preheated to 180 °C for 5 min. Subsequently, the dough cutouts were placed on a microwave-safe plate and baked for 25 min at 180 °C in convection mode. Then, the cookies (GL) were cooled at room temperature for 2 h. Each batch of cookie dough yielded eleven cookies, which were then transferred to an airtight container before being used for further experiments.

### 2.3. Analysis of the Cookie Dough

#### 2.3.1. Microscopic Analysis

The microstructure of cookie dough was studied under high-resolution confocal microscopy (model: Leica TSC SP8, Wetzlar, Germany) [15]. The dough for confocal microscopy was prepared by substituting water with Rhodamine B solution (40 µg/mL). This was done as Rhodamine B has been reported to help visualize and analyze protein structures within dough samples [16]. The remaining steps in the dough-making process were the same. The imaging was carried out in brightfield and CLSM modes. The excitation of the samples was carried out with a 543 nm laser, while the emitted fluorescence was detected in the 570–620 nm range. Imaging in the CLSM mode allowed a detailed analysis of the protein structure of the cookie dough.

#### 2.3.2. Moisture Content

A digital moisture analyzer was used to measure the cookie dough’s moisture content (model: PGB1MB Moisture analyzer, Wensar, India), operating with a halogen heating source at 180 °C. Then, the cookie dough (~2 g) was placed in the aluminum pan. The initial final weight of the dough was noted. Following that, the moisture content was calculated [17] using Equation (1).

(1) $$MC\;\left(\%\right)=\left(\frac{W_{I}-W_{F}}{W_{I}}\right)\times 100$$

where MC denotes the percentage of moisture content, W_I_ is the initial weight of the cookie dough sample, and W_F_ is the final weight of the cookie dough sample.

#### 2.3.3. FTIR

An FTIR spectrophotometer (Alpha-E; Bruker, Bremen, Germany) was used to record the FTIR spectra of the WWF dough in attenuated total reflectance (ATR) mode. The ATR crystal was made up of a ZnSe crystal. Approximately 2 g of the sample was directly spread over the ATR crystal. Each sample was scanned 25 times at a spectral resolution of 4 cm^−1^, whose average was reported as the sample spectrum. The samples were scanned in the 4000 cm^−1^–500 cm^−1^ wavenumber at room temperature (RT, 25 °C) [18].

Subsequently, the FTIR spectra were deconvolved using the “Gaussian function” after the baseline elimination by the subtraction method. The “Multiple Peak fit” tool in Origin Pro software (v9.1, Northampton, MA, USA) was used for performing peak fitting. Numerical deconvolution was employed to calculate the relative ratios of the three main bands (water region, amide I region, and starch region). The deconvolution was accepted if the R^2^ > 0.99 and χ^2^ < 0.001 [19].

#### 2.3.4. Texture Analysis

Texture analysis was performed using a texture analyzer (TA-HD plus, Stable Micro Systems, Godalming, UK). For spreadability, a load cell of 50 kg coupled with a 45° Perspex conical probe was used. The female Perspex cone was filled with the dough sample, and it was then secured in the cone holder. Once the penetration point was 2 mm from the bottom of the female cone, the male Perspex cone was allowed to continue penetrating the dough at a rate of 1 mm/s. At the same rate, the male cone returned to its former position. [20].

The stress relaxation (SR) test employed the Perspex conical probe, wherein the dough samples were loaded in the female Perspex cone. Thereafter, the male probe was allowed to penetrate the dough to a distance of 5 mm using a trigger force of 5 g (compression stage). Then, the dough samples were subjected to a constant compressive strain for 60 s (relaxation stage), followed by the retraction of the male probe to its original position. The pre-test, test, and post-test speeds were set to 1.0, 0.5 and 1.0 mm/s, respectively, and the F_0_ and F_60_ values were obtained. Subsequently, Equation (2) was used to calculate the percentage of SR (%SR) of the cookie dough samples [21].

(2) $$\%SR=\frac{F_{0}-F_{60}}{F_{0}}\times 100$$

where %SR is the percentage of stress relaxation, F_0_ is the maximum force at the end of the compression stage, and F_60_ is the residual force at the end of the relaxation stage.

#### 2.3.5. Impedance

The impedance profile of the dough samples was measured using an impedance analyzer (Impedance breakout board for Analog Discovery 2, Digilent, National Instrument, Austin, TX, USA). A pair of circular stainless-steel probes (diameter: 10 mm), placed at a distance of 1 cm, was used for the impedance spectroscopy. The electrode system was inserted into the dough, and the impedance profile was measured in the frequency range of 1 Hz and 1 KHz. During the measurement, the reference resistor of 1 MΩ was used for the analysis [22].

### 2.4. Analysis of the Cookies

#### 2.4.1. Surface Topology of Cookies

The surface topology of the cookies was analyzed using an AMscope Stereo Zoom microscope (SM-2TZ, AMscope, Irvine, CA, USA) attached to a microscope camera (MD500, AMscope, Irvine, CA, USA).

#### 2.4.2. Physical Dimension of Cookies

The width (W) of the baked cookies was measured using a digital Vernier caliper (Model: IP54 Metal case Digital caliper; Make: Advance, West Bengal, India), while the thickness (T) was measured using a digital micrometer (Model: EM025; Make: Digital Micrometre, Yuzuki, China). Subsequently, the spread ratio (SRA) of the cookies was expressed as the average width (W_Av_) divided by the average thickness (T_Av_) in Equation (3) [23].

(3) $$SRA=\frac{W_{Av}}{T_{Av}}$$

where SRA denotes the spread ratio of cookies, W_Av_ is the average width of cookies, and T_Av_ is the average thickness of the cookies.

#### 2.4.3. Moisture Content of Cookies

The moisture analysis of the cookies was performed using a digital moisture analyzer (Model: PGB1MB Moisture analyzer; Make: Wensar, India), operating with a halogen heating source at 180 °C. Before analysis, the cookies were pulverized, and approximately 2 g of the resulting powder was loaded into the moisture analyzer [24]. As with the cookie dough, the initial and final weights were recorded to calculate moisture content using Equation (1).

#### 2.4.4. FTIR of Cookies

The cookies were powdered and were used to determine FTIR spectra, following a similar procedure to that used for the cookie dough.

#### 2.4.5. Texture Analysis of Cookies

The hardness of the cookies was assessed via the three-point bending test (3-PBT) with the texture analyzer attached to a 50 kg load cell. For the experiment, a 3-point bending rig (HDP/3PB) was used. Then, the cookie was positioned on adjustable supports spaced 2 cm apart. Subsequently, compression force was applied to the cookie using the flexure attachment, which moved a distance of 10 mm after a trigger force of 5 g. The analysis was performed at a speed of 1 mm/s. The maximum peak force required to break the cookie with the flexure attachment was recorded as the cookie hardness.

A puncture test was performed used to determine the fracturability of the cookies. For the analysis, cookies were positioned on the platform and punctured using a 3 mm cylindrical stainless-steel probe (P/3) to a depth of 1 mm, with a trigger force of 5 g. The pre-test, test, and post-test speeds were set at 0.5 mm/s, 0.5 mm/s, and 1 mm/s, respectively. The highest force required for the probe to travel 1 mm was recorded as cookie fracturability [25].

### 2.5. Statistical Analysis

The experiments were carried out in pentaplicate (five replicates), and the results were reported as average ± standard deviation. Statistical analysis was conducted using IBM’s SPSS Statistics 20 (IBM Inc., Chicago, IL, USA). The significance difference was determined using a one-way analysis of variance (ANOVA), followed by a Tukey post hoc test. A significance level of (*p* < 0.05) was used to indicate a statistically significant difference between values.

## 3. Results and Discussion

### 3.1. Analysis of Dough

#### 3.1.1. Microscopic Analysis

##### Brightfield Microscopy

**Figure 1:**
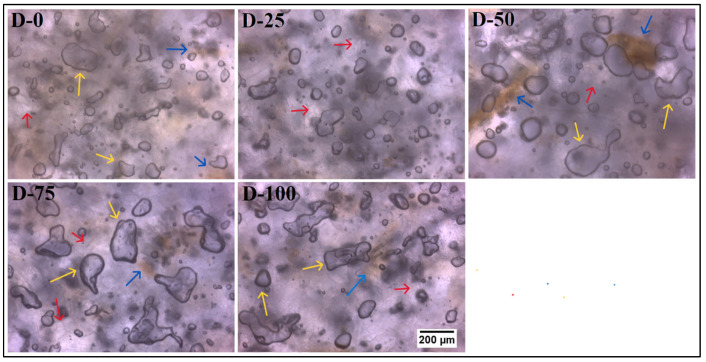
Brightfield micrographs of dough samples (magnification 10×). Starch particles (marked with red arrows), bran particles (marked with blue arrows), and water molecules (marked with yellow arrows).

The brightfield microscopic analysis (under 10× magnification) of dough samples provides valuable insights into the interactions between ingredients, gluten network formation, and the overall quality of the dough [26]. In our case, the brightfield micrographs have revealed the presence of three distinct components—starch, bran, and the distribution of water molecules (Figure 1). In the micrographs, the presence of translucent structures (marked with red arrows) suggests the presence of evenly dispersed starch particles. The WWF dough exhibited the presence of bran particles (yellow-brown colored) (marked with blue arrows). Furthermore, the enclosed structures within the sample can be regarded as distributed water molecules (marked with yellow arrows) [27]. This suggests that an increasing concentration of oleogel resulted in a discernible alteration in water distribution. The dough samples D-0 and D-25 exhibited a uniform water distribution with minor water clusters, indicating that the starch matrix absorbed water molecules. However, with the increment of oleogel concentration in D-50 and D-75, the water agglomerates observed were larger in comparison to the former samples. Intriguingly, in sample D-100, the water agglomerates appeared smaller than those observed in D-50 and D-75 but displayed a non-uniform distribution. The presence of the oleogel in high concentrations in D-100 can account for the non-uniform distribution of the water phase. It has been reported that oleogel can affect the distribution and organization of water molecules within the dough by altering the interactions between water and other dough components, such as starch and proteins [28].

##### Confocal Laser Scanning Microscopy (CLSM)

**Figure 2:**
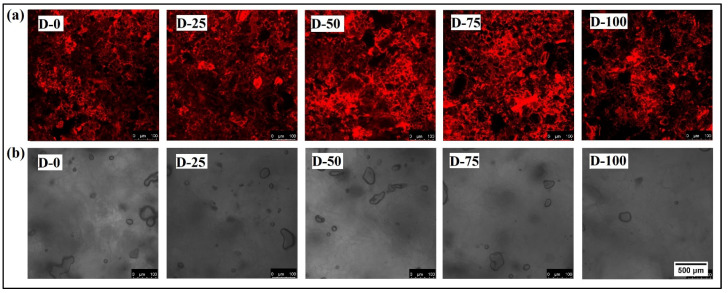
(**a**) CLSM micrographs of the cookie dough samples, and (**b**) brightfield micrographs of cookie dough samples (magnification 20×).

CLSM is a powerful imaging technique that uses laser light to create high-resolution images of samples. The Rhodamine B-loaded dough was analyzed under high-resolution confocal microscopy (magnification: 20×) for detailed protein analysis (Figure 2a) [16]. The micrographs of D-0 and D-25 showed identical protein distributions, which can be attributable to the fact that increasing the small amount of oleogel had no discernible impact on the protein network formation. In D-50, an optimal distribution of proteins was observed, likely due to the balanced combination of oleogel and butter in a 1:1 ratio. Similar micrographs were observed in D-75, indicating comparable protein distribution. Conversely, in D-100, uneven distribution of the protein network due to aggregation was observed. Based on these findings, it can be inferred that increasing the content of oleogel up to D-75 promotes improved protein network formation [29].

Additionally, the brightfield micrographs (Figure 2b) from the CLSM microscope revealed the presence of starch granules. These granules appeared as bright, densely packed structures, indicating their abundance within the dough. The starch granules were evenly dispersed throughout the sample, forming a matrix-like structure. Furthermore, the CLSM images provided insights into the distribution of water molecules within the whole wheat dough. Water appeared as a background medium, surrounding and permeating the different components of the dough.

#### 3.1.2. Moisture Analysis

**Figure 3:**
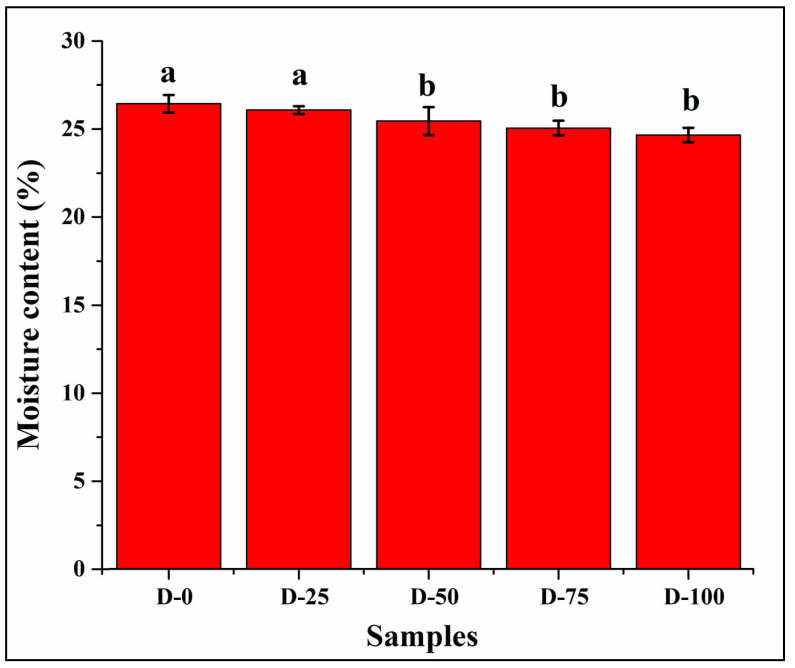
Moisture analysis of cookie dough samples. Mean values with different letters above columns of the same colored bar are statistically different (α = 0.05).

The moisture content in a dough sample plays a crucial role in determining its physical, chemical, and rheological properties, which are essential for dough handling and shaping [30]. Moreover, moisture content impacts dough hydration, which, in turn, helps to fine-tune the texture, flavor, and appearance of the cookies. Therefore, measuring and controlling the moisture content in a dough sample is critical for optimizing the baking process and ensuring consistent and high-quality cookies. Typically, the dough samples have a high moisture content (20–25%) [31] (Figure 3). Among all the samples, the control dough sample (D-0) had the highest moisture content. D-0 (26.42 ± 0.49) and D-25 (26.06 ± 0.22) dough samples showed statistically similar moisture content values (*p* > 0.05). As the proportion of oleogel was increased, the moisture content of the samples decreased significantly. Samples D-50, D-75, and D-100 had lower moisture content values of 25.43 ± 0.79, 25.04 ± 0.42, and 24.63 ± 0.52, respectively. The decline in moisture content can be attributed to the reduction in butter content, which was approximately 18% moisture. Therefore, the decrease in butter content resulted in a reduction of moisture content in the dough samples [32].

#### 3.1.3. FTIR Analysis of Dough

**Figure 4:**
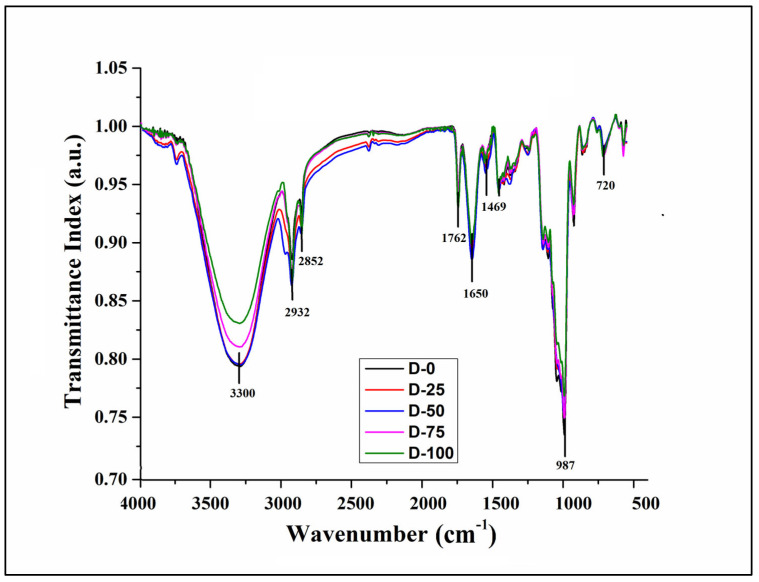
FTIR spectra of the dough samples.

FTIR (Fourier transform infrared) spectroscopy technique was utilized to obtain the FTIR spectra of cookie dough samples in the spectral range of 4000 cm^−1^ to 400 cm^−1^. The region between 1400 cm^−1^ and 700 cm^−1^ is known as the fingerprint region, and the rest between 1500 cm^−1^ and 4000 cm^−1^ is called the functional group region. In our dough samples, the major peaks detected in the spectrum were observed at wavenumbers of 3300, 2932, 2852, 1762, 1650, 1469, 987, 922, and 720 cm^−1^ (Figure 4). The broadband peak centered at around 3300 cm^−1,^ followed by the peaks at 2932 cm^−1^ and 2852 cm^−1,^ are related to the stretching vibration of hydroxyl groups (OH). Another peak observed between 1762 cm^−1^ and 1600 cm^−1^ can be due to the stretching vibration of carbonyl (C=O) groups [33,34]. Further, the spectral region between 1400 cm^−1^ and 1550 cm^−1^ represents the Amide II region. They include molecular vibrations associated with N-H bending, C-N, and C-C bending of gluten proteins [35]. Subsequently, the region between 1200 cm^−1^ and 1340 cm^−1^ represents the N-H bending and C-N bending regions. Further in the spectrum, the backbone vibrations of C-O, C-N, and C-C bonds were responsible for spectral regions between 900 cm^−1^ and 1200 cm^−1^. At last, the peak observed near 700 cm^−1^ corresponds to the bending vibration of the hydroxyl group (COH), which may be related to the presence of free water molecules. Three prominent peaks found near the water, protein (Amide I), and starch region play a significant role in determining the texture and quality of the dough. To gain a deeper understanding, we have further deconvoluted them further for analysis with the help of the Gaussian function [33].

**Figure 5:**
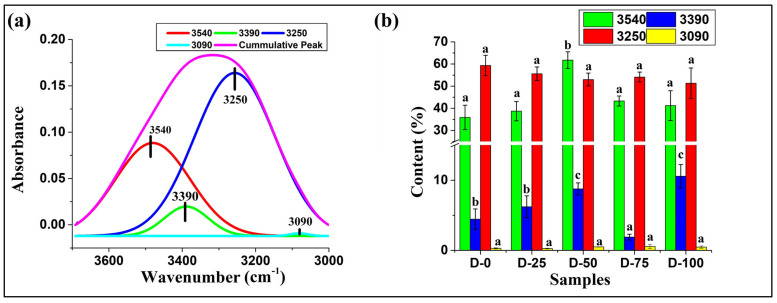
Analysis of the water structure via FTIR spectroscopy. (**a**) Deconvoluted bands of the water region (3800 cm^−1^–3000 cm^−1^), and (**b**) variation in the area under the curve (AUC) of the peaks obtained in the water region. Mean values with different letters above columns of the same colored bar are statistically different (α = 0.05).

The physicochemical attributes of water, such as its content, distribution, and motility, as well as its interplay with other components within the food matrix, have a pronounced influence on the stability and functional characteristics of foods based on starch [36]. Walrafen et al. formulated a theoretical framework for the structure of water. They posited that the OH spectral band results from the overlapping of five distinct bands situated at wavenumbers 3090, 3220, 3393, 3540, and 3625 cm^−1^ [37]. The peak observed at 3090 cm^−1^ is attributed to the Fermi resonance of the overtone of the OH plane bending with the O-H vibration of a strongly hydrogen-bonded water structure. Subsequently, the peak observed at 3220 cm^−1^ is due to strongly bonded O-H interactions. Additionally, it exhibits Fermi resonance with the O-H vibration of a strongly hydrogen-bonded water molecule in the 3215 cm^−1^ to 3220 cm^−1^ region [38]. The peak at 3390 cm^−1^ corresponds to weakly bound water interaction, and the peak observed at 3540 cm^−1^ is attributed to the symmetric O-H vibrations of a non-hydrogen-bonded or free water molecule. Further, the peak observed at 3620 cm^−1^ is due to the asymmetric vibration of a non-hydrogen-bonded or free-water molecule. In our case, 4 distinct peaks at 3540 cm^−1^ (peak 1), 3390 cm^−1^ (peak 2), 3220 cm^−1^ (peak 3), and 3090 cm^−1^ (peak 4) were observed in the water region (Figure 5a). The peak at 3625 cm^−1^ was absent in all of the samples. A peak position shift (approximately ±10–20 cm^−1^) was noted for all the peaks obtained during the deconvolution process. This shift may be attributed to the variation in dough composition. Among the 4 peaks, only 2 peaks were dominant: 3540 cm^−1^ (Peak 1) and 3250 cm^−1^ (Peak 3). Peak 1 corresponds to the symmetric O-H vibrations of the free water molecules [19]. All the samples, except D-50, showed similar areas under the curve (AUC) values for peak 1 (*p* > 0.05). In comparison to others, the AUC of D-50 was considerably high. This suggests that in D-50, the quantity of free water molecules was considerably higher than in other samples. Peak 2 corresponds to the weakly bound water molecules. The AUCs of D-0 and D-25 had similar values (*p* > 0.05) and were lower than the AUCs of D-50 and D-100, which also had similar value (*p* > 0.05). D-75 showed the lowest AUC value, suggesting the presence of a meager quantity of weakly bound water molecules. Similarly, peak 3 corresponds to the strongly bonded water molecules. Additionally, it exhibits Fermi resonance with the O-H vibration of strongly bonded water molecules in regions between 3215 cm^−1^ and 3220 cm^−1^ [19]. All the samples showed similar AUC values for peak 3 (*p* > 0.05). Subsequently, peak 4 corresponds to the overtone of the OH plane bending, coupled with the O-H vibration, exhibiting Fermi resonance in strongly hydrogen-bonded water. This peak was quite negligible as the values were quite low compared to the other peaks and their AUC values were similar for all the samples (*p* > 0.05).

**Figure 6:**
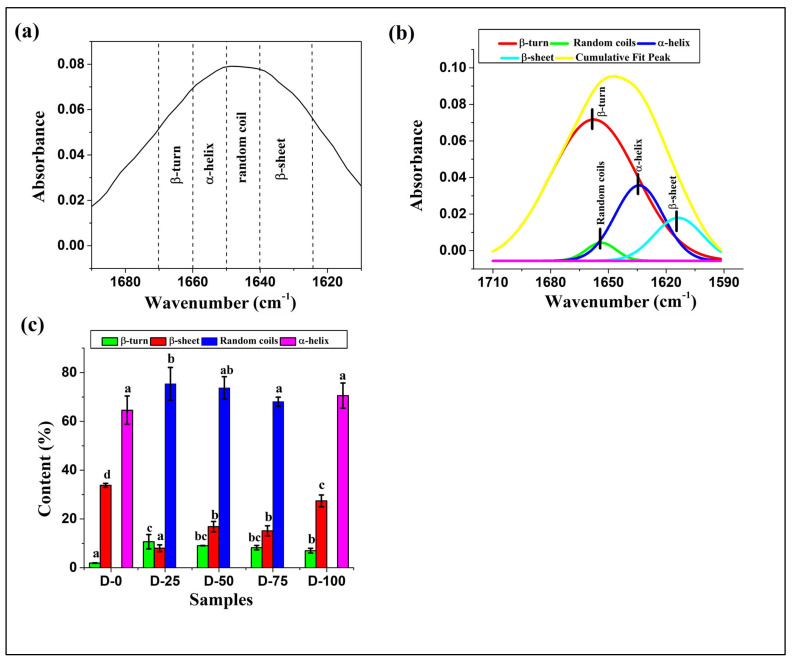
Analysis of the protein structure via FTIR spectroscopy. (**a**) The Amide I region containing different protein secondary structures at different wavelengths, (**b**) deconvoluted bands of the Amide I region (1600 cm^−1^–1700 cm^−1^), and (**c**) variations in the area under the curve (AUC) of different secondary structures obtained in the Amide I region. Mean values with different letters above columns of the same colored bar are statistically different (α = 0.05).

The Amide I spectral region in FTIR analysis is a critical region for characterizing secondary structures of proteins. This is of paramount significance because the secondary structure of protein plays a vital role in determining how proteins interact and function [39]. The analysis focused on the four sections of the amide I band in FTIR spectra (Figure 6b). Firstly, the region between 1660 and 1670 cm^−1^ and 1694 cm^−1^ is associated with β-turn conformation. Then, the peak between 1650 and 1660 cm^−1^ corresponds to α-helix conformation, while the band located at 1640 and 1650 cm^−1^ corresponds to the random coil conformation of the proteins. Lastly, the band between 1624 and 1640 cm^−1^ and 1681 cm^−1^ are associated with β-sheet conformation [40]. In our samples, secondary structures could be related to the conformations β-turn, α-helix, random coil, and β-sheet. All the samples had β-turn and β-sheet, but only a select number of samples showed α -helix and random coil (Figure 6c). Recent studies have reported that α -helix and β-sheet are the most stable secondary structures, whereas β-turn and random coil indicated a more flexible protein structure [41]. D-0 and D-100 samples exclusively displayed α -helix structures and have high AUC for β-sheet, suggesting the protein network in these samples is more stable than in others. It can be speculated that only butter (in the case of D-0) and only oleogel (in the case of D-100) provided better stability over other samples which had a blend of both (butter and oleogel). D-25, D-50, and D-75 samples exclusively displayed random coil structures and had high AUC for β-turns, indicating the formation of flexible protein networks in these samples. Overall, it can be assumed that when butter and oleogel are combined in different proportions, the dough becomes flexible.

**Figure 7:**
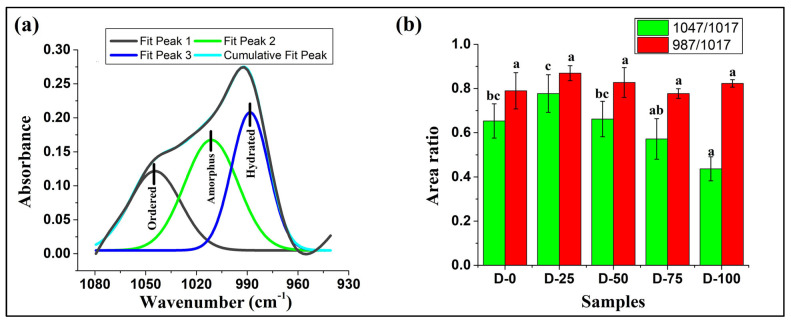
Analysis of the starch structure via FTIR spectroscopy. (**a**) Deconvoluted bands of the starch region (1070 cm^−1^–950 cm^−1^), and (**b**) variations in the area under the curve (AUC) structures obtained in the starch region. Mean values with different letters above columns of the same colored bar are statistically different (α = 0.05).

García-Valle et al. (2021) reported that the spectral range between 1070 cm^−1^ and 950 cm^−1^ depicts the starch fingerprint region and its molecular organization [42]. The absorption peak at the wavenumber of ~995 cm^−1^ is linked to hydrated crystalline starch, formed due to the hydrogen bonding of the hydroxyl groups of the starch macromolecules. Subsequently, the band at 1017 cm^−1^ is associated with an amorphous region of starch. Lastly, the absorption band at 1047 cm^−1^ corresponds to the ordered crystalline structure of starch (Figure 7a). Capron et al. (2007) reported that the ratio of 1047 cm^−1^ to 1017 cm^−1^ (ratio 1) is used to determine the alignment of helices within the short-range order influenced by the presence of water molecules that are bonded to the starch molecules. The ratio of 995 cm^−1^ to 1022 cm^−1^ (ratio 2)) is a widely used parameter for assessing starch’s double helices and short-range structure (Figure 7b) [43]. In ratio 1, samples D-0, D-25, and D-50 had similar AUC values (*p* > 0.05). AUC for D-75 and D-100, which had similar values (*p* > 0.05), was lower than the other 3 samples (*p* < 0.05). As the concentration of oleogel increased, it promoted the oleogel layer around the starch granules resulting in the formation of an amylose–lipid complex. This layer may have prevented the amylose double-helix structures in the starch chains from reorganizing, resulting diminution of the ordered structure [19]. There were no statistical differences between the AUC values of ratio 2 (*p* > 0.05), suggesting that there was a negligible rearrangement of double helices due to the replacement of butter with oleogels [44].

#### 3.1.4. Texture Analysis of Dough

**Figure 8:**
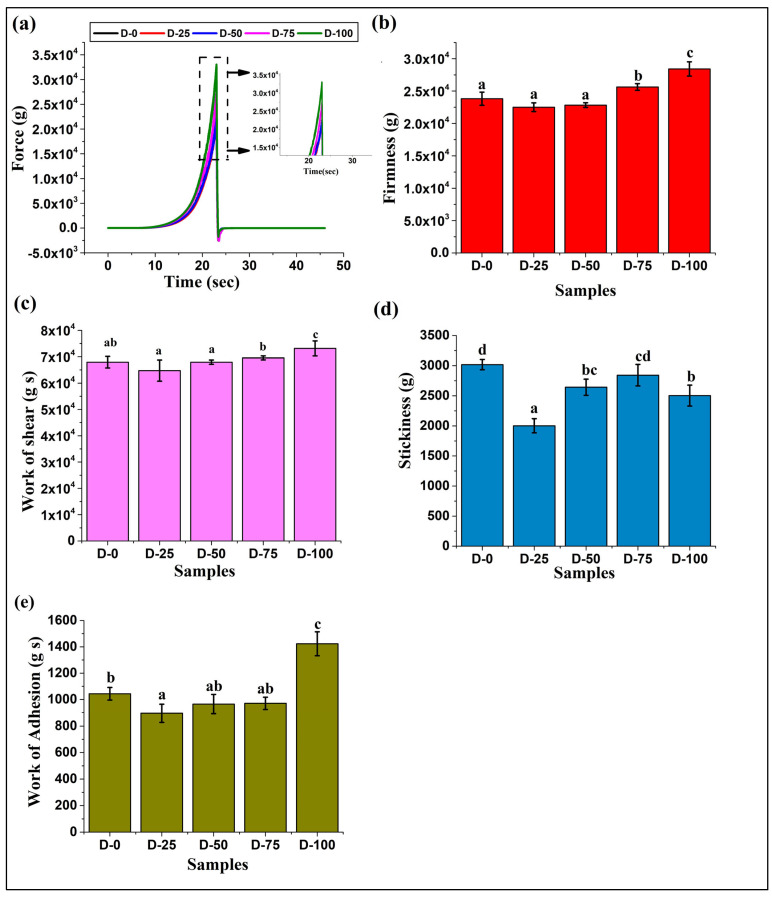
(**a**) Spreadability profile of cookie dough parameters of cookie dough. Spreadability parameters: (**b**) firmness, (**c**) work of shear, (**d**) stickiness, and (**e**) work of adhesion. Mean values with different letters above columns of the same colored bar are statistically different (α = 0.05).

According to Suriya et al. (2017), dough with greater spreadability is generally considered more desirable. As a result, it is essential to comprehend the effects of the composition of dough on the extent of cookie dough spread [45]. Several derived parameters, such as firmness, stickiness, work of shear, and work of adhesion, were determined following the testing of the samples (Figure 8a).

The firmness of a cookie dough sample refers to its ability to resist deformation under an applied force. It is an important texture attribute that can impact consumer perception of product quality, freshness, and overall satisfaction [46]. The analysis of the dough samples showed that D-0, D-25, and D-50 had similar firmness values (*p* > 0.05), which were lower compared to the remaining samples. This may be due to their higher fat content in D-75 and D-100, which contained a relatively higher concentration of butter. Fat has a tenderizing effect on cookie dough. It coats the gluten proteins in the dough, effectively shortening the strands and reducing gluten development [47]. D-100 had the highest firmness value (32,412.17 ± 1103.02 g), which might be due to the higher and uneven distribution of gluten in the dough, as seen in the CLSM micrograph (Figure 2). It is also possible that because oleogel has less fat than butter, there was a decrease in tenderizing, which in turn caused the firmness values to be higher [48].

When a force is applied to a dough, it causes the dough to deform and change its shape. The force that causes the deformation of the dough is called shear force, and the work done by this force is known as the work of shear [49]. It can be expected that to deform the samples with increased stiffness, a higher level of shear force will be necessary. The work of shear and firmness are often found to have a strong correlation [50]. The results from our study also indicated that the work of shear values displayed a pattern similar to that of the firmness value. Based on this observation, it can be inferred that higher shear work leads to less spreadable cookie dough. It was observed that the work of the shear of D-0 was similar to that of D-25, D-50, and D-75. However, the work of shear of D-100 was the highest (*p* < 0.05).

The stickiness of the dough is a tactile property that describes the dough’s texture and its ability to maintain its shape without excessive adherence, represented by the highest negative peak in the spreadability profile (Figure 8a) [51]. Gliadin protein plays an essential role in the stickiness of dough samples. D-0 showed the highest stickiness among all the samples, which may be attributed to the presence of gliadin protein in α-helix conformation [52]. This was evident from the FTIR spectroscopy, where a high proportion of α-helix was observed in D-0, resulting in dough formation with the highest stickiness (Figure 6c). D-25 showed the lowest stickiness value, possibly due to the high proportion of random coil conformation of gliadin (Figure 6c) [53].

Work of adhesion showed a similar pattern to that of the stickiness values except for D-100, which was significantly higher than other samples. The presence of very high amounts of oleogel can be the reason for this. It has been previously reported that oleogels form strong interactions with the interacting surfaces, thereby promoting adhesion [54].

**Figure 9:**
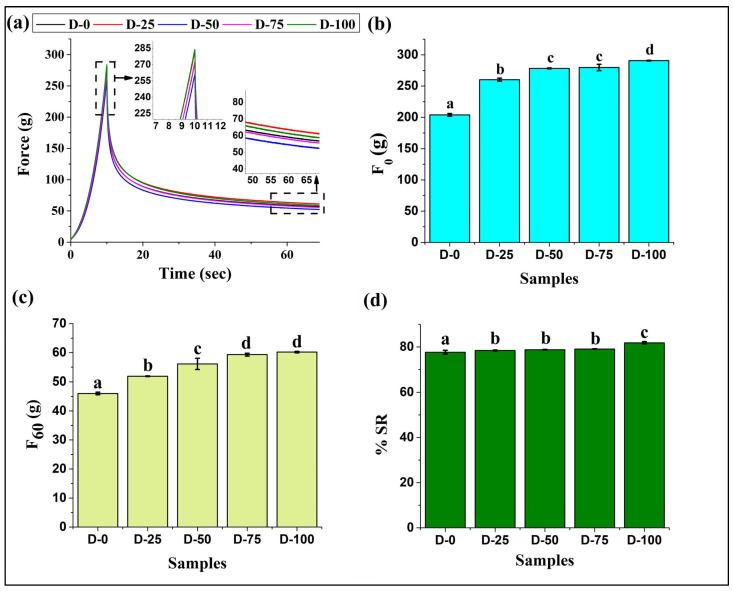
(**a**) Stress relaxation profile of cookie dough. Stress relaxation parameters (**b**) F_0,_ (**c**) F_60,_ and (**d**) %SR. Mean values with different letters above columns of the same colored bar are statistically different (α = 0.05).

Stress relaxation occurs in two phases: Initial rapid relaxation occurs immediately (0.1–0.5 s) after the dough is subjected to stress or deformation. During this phase, the dough structure adjusts and redistributes to accommodate the applied force. Rapid relaxation occurs within a short period, usually within the first few seconds after the stress is applied. Long-term relaxation (>10 s) is a phase in which the stress continues to decrease but at a slower rate than the initial phase. The long-term relaxation process occurs over an extended period, depending on the specific characteristics of the dough [55]. Various factors influence stress relaxation, including formulation, ingredient properties, processing conditions, and storage conditions. The firmness of the cookie dough samples was obtained from the maximum force (F_0_ values) under strain. The results showed that D-0 had the lowest firmness for the dough sample with an F_0_ value of 203.97 ± 2.118 g. As the proportions of oleogel increased, the firmness increased significantly in D-25 and D-50 (*p* < 0.05). The firmness of D-75 was similar to that of D-50 (*p* > 0.05), while the firmness of D-100 was the highest (*p* < 0.05). This suggests that the proportional reduction of butter positively impacts the firmness of the samples. Butter, which is 80% fat, has a tenderizing effect. Therefore, as the concentration of oleogel (which likely contains less fat than butter) increased, the tenderizing effect decreased, resulting in increased firmness values [47]. During the relaxation phase, the force values declined to a basal level, referred to as the residual force (F_60_), when the probe was held in the same position. F_60_ is also known as dough elasticity. The results indicate that the F_60_ values exhibited a similar pattern to the F_0_ values of the dough samples. The %SR observed for the dough samples was higher than 75%, suggesting a predominant fluidic behavior as there was an increase in oleogel concentration [56] (Figure 9).

#### 3.1.5. Impedance Analysis

**Figure 10:**
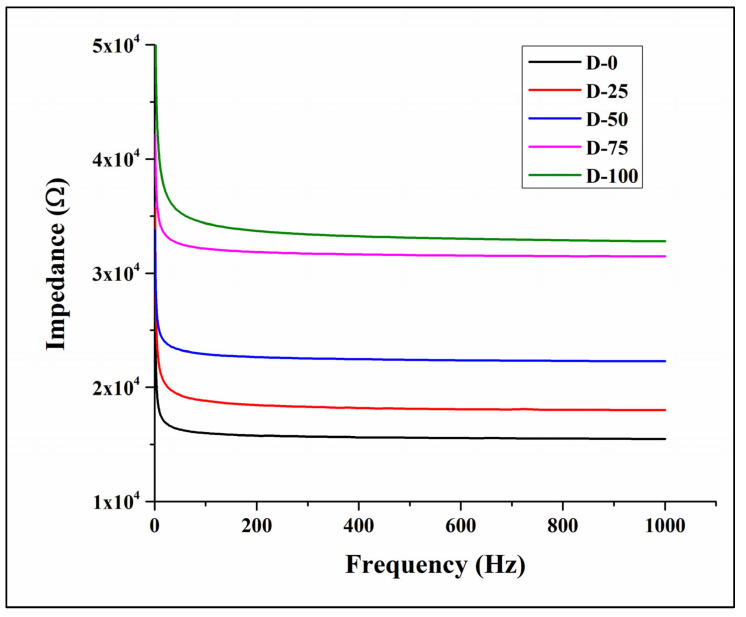
Impedance profile of cookie dough.

Electrical impedance analysis (EIA) of dough is a technique for analyzing the electrical properties of dough. EIA involves passing an alternating current (AC) through a dough sample and measuring the resulting impedance, which is the resistance to the flow of electrical current. The electrical properties of dough change during processing and can affect the texture and quality of the final product [57]. In our case, dough sample D-0 exhibited the lowest impedance values compared to the rest. The impedance increased as the proportions of oleogel increased. This might be speculated due to the non-conducting nature of the oleogels [58]. Interestingly, as the oleogel content increased from 50% to 75%, there was a considerable increase in the impedance values in D-75 compared to D-50. This might be due to the presence of low proportions of weakly bound water molecules in D-75, which was evident from FTIR spectroscopy (Figure 5b). Recent studies have reported that these weakly bounded water molecules have a higher dielectric constant [59]. The dielectric constant is inversely proportional to impedance. In other words, as D-75 had a lower proportion of weakly bound water, the dielectric constant of D-75 was higher, and hence a considerable rise in impedance was observed. Among all the samples, D-100 had the highest impedance. This rise in impedance may be attributed to the reduction in the moisture content of the dough [60] (Figure 10).

### 3.2. Analysis of Cookies

#### 3.2.1. Visual Appearance of Cookies

**Figure 11:**
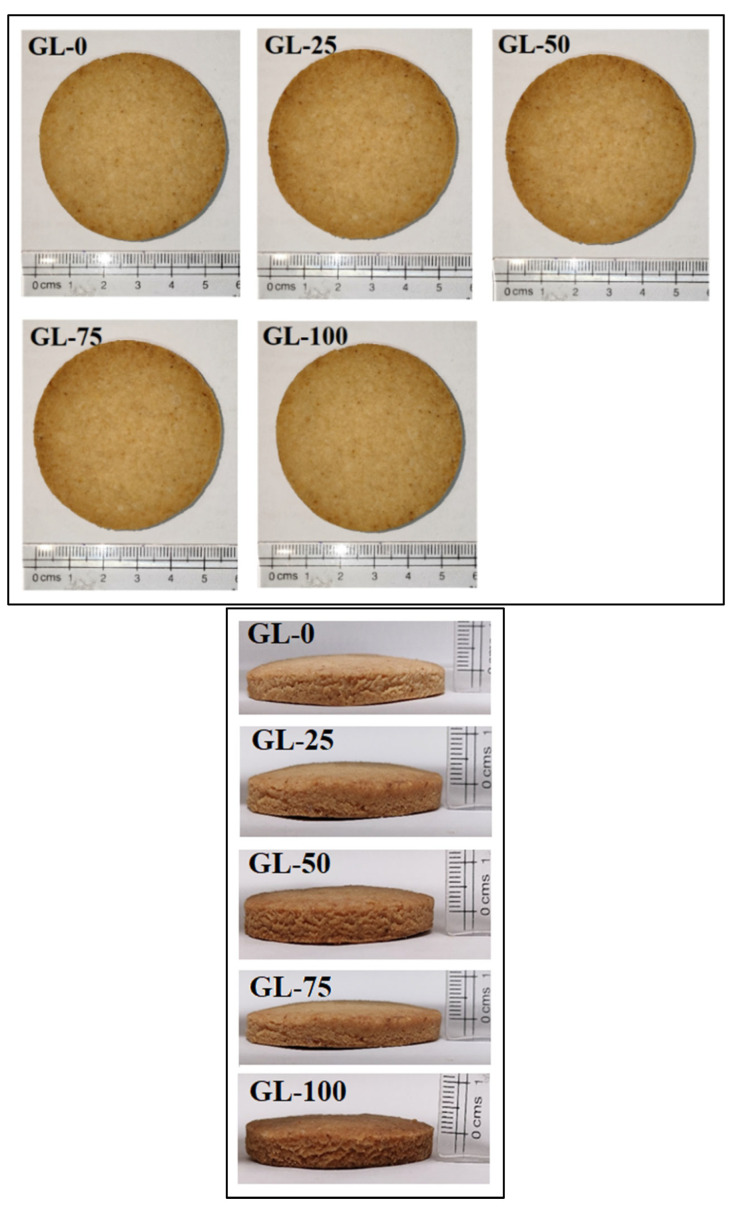
Visual appearance of the cookies.

The baked cookies had a light brown color, which can be attributed to sugar crystallization during the baking process [61]. The edges of the baked cookies displayed signs of browning, suggesting that the process of browning initiates from the periphery and gradually extends toward the center. This can possibly be attributed to a phenomenon known as the Maillard reaction, which occurs between amino acids and reduces sugars in food when exposed to heat. When cookies are baked, the high temperature causes the Maillard reaction between the proteins (amino acids) present in the dough and the sugars. The reaction proceeds in several stages and produces a variety of flavor compounds and brown pigments, giving the cookies their characteristic aroma, taste, and color [62]. As the edges of the baked cookies were brown in color, it can be expected that the cookies were baked to an optimal extent. All the cookies displayed a light-colored center even though the edges were brown. There was no change in the visual appearance of cookies as the oleogel concentration varied (Figure 11).

#### 3.2.2. Surface Topology of Cookies

**Figure 12:**
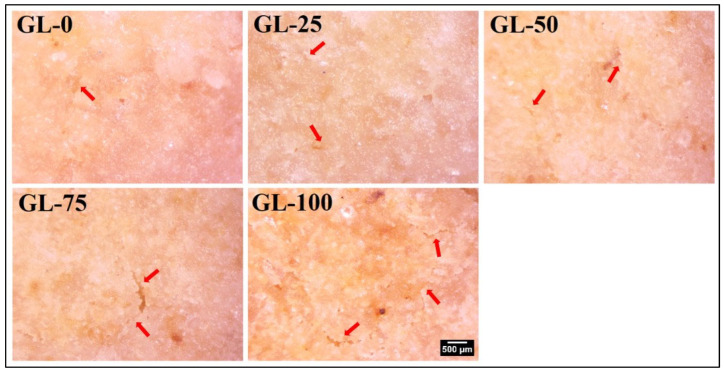
Surface topographs of cookies. Red arrows represents crack in the cookies.

The surface topology of cookies plays a crucial role in the overall texture and taste of the final product. The appearance of the cookie’s surface can influence its visual appeal, making it more or less appealing [63]. An uneven surface micrograph with cracks was observed (marked with red arrows) (Figure 12). The control sample GL-0 exhibited minimal surface cracks. However, at higher oleogel concentrations, there was a noticeable increase in surface cracks [64]. This can be reasoned to fat migration during the baking process of the cookies. During baking, the molten fat migrates within the dough, thereby coating the flour particles and forming a barrier between the dough and the surrounding air. This barrier helps to retain moisture within the dough, leading to a softer and more tender texture in the finished cookies. However, an excess amount of fat or oil in the dough can lead to an imbalance in the dough’s structure. As the fat melts and migrates, it acquires a fluid-like consistency, which can result in the dough spreading too much during baking. This excessive spreading can cause the dough to lose its structural integrity and result in surface cracks [65].

#### 3.2.3. Physical Dimension

**Figure 13:**
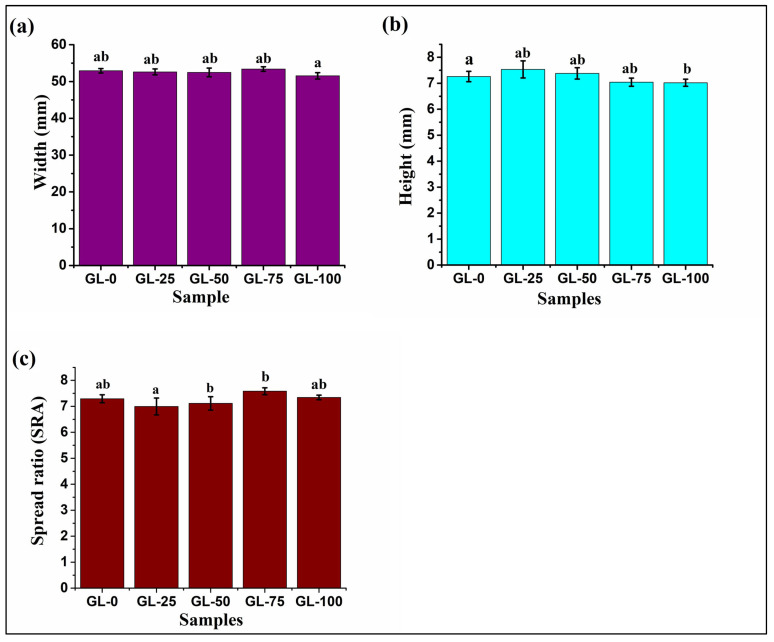
Dimensions of the cookies, (**a**) Width, (**b**) Height, and (**c**) Spread ratio. Mean values with different letters above columns of the same colored bar are statistically.

Dimensions such as height, width, and spread ratio were measured for each cookie sample. The spread ratio and the baking duration determine the cookie’s width (Figure 13). The width of the cookie sample ranged between 51 and 54 mm. There were no significant changes in the widths of GL-0, GL-25, and GL-50 for the prepared cookies (*p* > 0.05). It can be speculated that increasing the concentration of oleogel had no effect on cookies till the ratio of oleogel to butter was 1:1. However, GL-75 and GL-100 exhibited significantly different values (*p* < 0.05). The height of the cookie samples ranged between 7.0 and 7.6 mm. Regarding height, GL-0 and GL-50 were similarly valued (*p* > 0.05) and were higher than GL-75 and GL-100, which were also similarly valued (*p* > 0.05). GL-25 had the highest height among the samples. Subsequently, for SRA, GL-25 and GL-50 had similar values (*p* > 0.05) and were lower than GL-0 and GL-100, which also had similar values (*p* > 0.05). Among all, GL-75 and GL-100 displayed the highest SRA. Recent studies have reported that the dilution effect of oleogels on gluten could be responsible for the observed result. The spread ratio of cookies may have increased, and the thickness may have decreased as a result of this effect’s increase in fluidity [66]. Overall there were minute changes in the physical dimension of cookies when different proportions of oleogels were used.

#### 3.2.4. Moisture Content of Cookies

**Figure 14:**
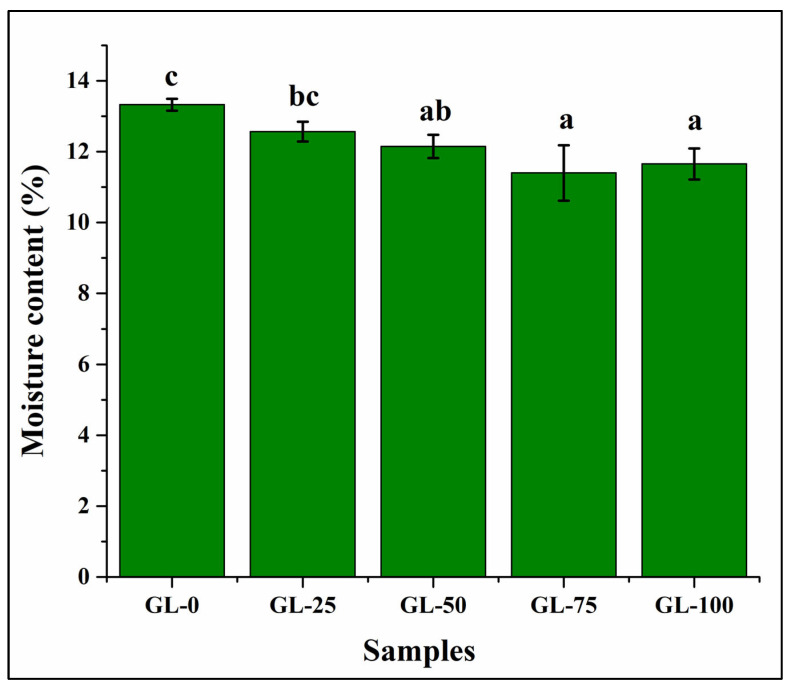
Moisture content (%) of the cookie sample. The alphabets above columns of the same color bar denote statistically significant (*p* < 0.05) differences.

Moisture control is a critical aspect that affects the quality and shelf life of cookies. The moisture content of cookies influences their texture, flavor, and appearance. An appropriate amount of moisture is essential to ensure that the cookies are not too dry or too moist, which can negatively impact their sensory attributes [67]. The moisture content of GL-0 was the highest, measuring 13.33 ± 0.17%. As the butter content decreased, the moisture content gradually decreased (Figure 14). The moisture content decreased as the proportion of butter in the cookies decreased. It ranged between 13% and 15%. The moisture content of a cookie is affected by the amount of moisture present in the dough from which it is made. As the quantity of butter in the dough declined, a corresponding decrease in the moisture level of the cookies was observed [32].

#### 3.2.5. FTIR of Cookies

**Figure 15:**
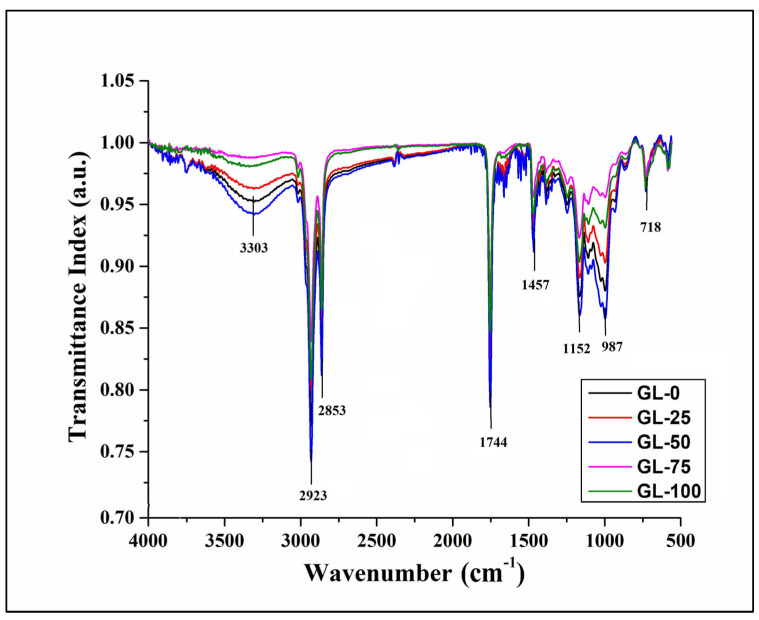
FTIR spectra of cookie samples.

The FTIR spectra of WWF cookies with change in concentration of oleogel are illustrated in Figure 15. Analyzing the spectra from the highest to the lowest wavenumbers, the major peaks were obtained at 3303, 3009, 2922, 2853, 1744, 1457, 1289, 1152, 987, and 718 cm^−1^. The broad peak at 3303 cm^−1^ corresponds to the NH stretching of water molecules, whereas the spectral range between 3000 cm^−1^–2500 cm^−1^ is associated with CH stretching [68]. Another peak at 1744 cm^−1^ and 1457 cm^−1^ corresponds to the C=O stretching of amides and NH stretching of the methyl group. Several peaks were found between 1400 and 900 cm^−1^. These peaks could be ascribed to carboxylic acids, ethers, and alcohols, corresponding to CH_2_OH-related stretching vibrations modes. Lastly, the broad bands in the region of 800 to 600 cm^−1^ were due to the C=C bond bending vibrational modes of the aromatic ring of glucose pyranose [69].

#### 3.2.6. Texture Analysis of Cookies

**Figure 16:**
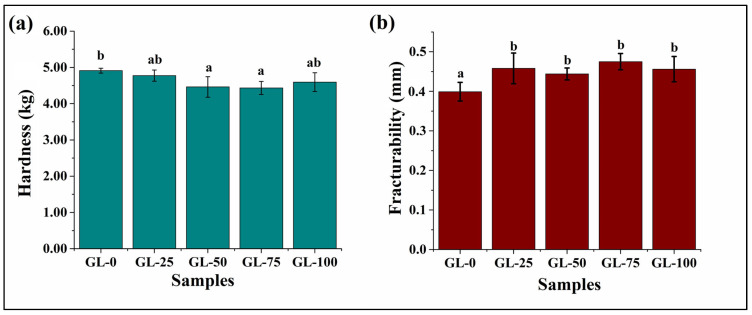
Texture parameters of cookies. (**a**) hardness, and (**b**) fracturability. Mean values with different letters above columns of the same colored bar are statistically.

Texture analysis is a method used to evaluate the physical properties of cookies. It involves assessing various textural attributes such as hardness, chewiness, crispness, and brittleness. These measurements provide insights into the sensory experience and quality of the cookies [70]. For the analysis of the cookie samples, we have taken two major texture parameters: hardness and fracturability (Figure 16). Hardness refers to the ability of cookies to withstand biting or breaking, indicating their level of resistance. The hardness of the cookie samples ranged between 4400 and 4800 g. Cookies with similar hardness values have been reported in IF Bolarinwa et al. [71]. Among them, GL-0 had the highest hardness values. This can be attributed to the fat structure in butter, which is more crystalline and rigid than the oleogels [72]. As the oleogel content increased. Fracturability describes how easily a cookie breaks or fractures. There were no significant differences in the fracturability of the cookie samples, except GL-0 (*p* < 0.05). This means that the inherent fracturability of cookies can be modified by using oleogel to replace butter. Overall replacing butter with varied proportions of oleogel did not affect the cookie texture to a great extent.

## 4. Conclusions

This investigation analyzed the results of replacing butter in cookies with a novel soy wax/rice bran oil oleogel. The oleogel was incorporated into the dough at four varied proportions, namely 25%, 50%, 75%, and 100%, by replacing butter. Characterization of cookie dough and cookies was conducted via various techniques to evaluate the overall effects of increasing the concentration of oleogel. The prepared cookie dough made with whole wheat flour displayed a soft and flexible texture yet was prone to crumbling. This particular texture is considered desirable for cookie dough. Moisture analysis of dough samples exhibited a consistent decrease, a trend that was further supported by the observed rise in impedance values. Confocal micrographs revealed a consistent protein network. However, incorporating higher percentages of oleogel (>75%) had an impact on the interconnectivity of proteins within the dough matrix. To analyze the changes in functional groups and molecular bonds within the dough matrix, FTIR analysis was performed. In the case of D-50, balanced water structures, as well as protein conformations, were observed, which was further supported by the CLSM and BFM micrographs. For the starch region, the ratio of 987/1017 showed negligible rearrangement of double helices. The firmness of the dough samples increased significantly as the oleogel content increased. The stress relaxation of the cookie doughs was >75%, indicating that the samples had a predominant fluidic behavior. The cookies showed a brown periphery and light-colored center because of Maillard’s reaction. Surface topography revealed an increase in surface cracks as the proportions of oleogel were increased. Moisture content ranged between 13% and 15%, which had a similar trend as cookie dough. However, minute changes were observed in dimensions as well as in the texture of the cookies. The study concludes that it is feasible to replace butter with SW/RBO oleogel up to 50% without significantly compromising the properties of dough and cookies. It was found that the cost of preparation of each batch was USD 0.862 and each batch yielded 11 cookies, hence the price per cookie was USD 0.078. However, the price per cookie could be brought down significantly when the same would be produced in higher quantities.

## Figures and Tables

**Table 1:** Composition of cookies.

Sample	Composition (g)							Weight of Dough Cutouts (Avg ± SD) (g)
	WWF	Butter	Oleogel	Sugar	Water	Salt	Baking Soda	
D-0	100.00	40.00	0	40.00	25.00	1.00	3.00	15.40 ± 0.84
D-25	100.00	30.00	10.00	40.00	25.00	1.00	3.00	15.60 ± 0.55
D-50	100.00	20.00	20.00	40.00	25.00	1.00	3.00	15.20 ± 0.45
D-75	100.00	10.00	30.00	40.00	25.00	1.00	3.00	15.20 ± 0.84
D-100	100.00	0	40.00	40.00	25.00	1.00	3.00	15.40 ± 0.45

## Data Availability

The data presented in this study are available on request.
